# Genome-wide identification of the *Phaseolus vulgaris* sRNAome using small RNA and degradome sequencing

**DOI:** 10.1186/s12864-015-1639-5

**Published:** 2015-06-02

**Authors:** Damien Formey, Luis Pedro Iñiguez, Pablo Peláez, Yong-Fang Li, Ramanjulu Sunkar, Federico Sánchez, José Luis Reyes, Georgina Hernández

**Affiliations:** Centro de Ciencias Genómicas, Universidad Nacional Autónoma de México (UNAM), Av. Universidad 1001, Cuernavaca, 62210 Morelos Mexico; Departamento de Biología Molecular de Plantas, Instituto de Biotecnología (UNAM), Av. Universidad 2001, Cuernavaca, 62210 Morelos Mexico; Department of Biochemistry and Molecular Biology, Oklahoma State University, Stillwater, OK 74078 USA

**Keywords:** *Phaseolus vulgaris*, microRNAs, phasiRNAs, Degradome, Nodules, Legumes, Common bean

## Abstract

**Background:**

MiRNAs and phasiRNAs are negative regulators of gene expression. These small RNAs have been extensively studied in plant model species but only 10 mature microRNAs are present in miRBase version 21, the most used miRNA database, and no phasiRNAs have been identified for the model legume *Phaseolus vulgaris*. Thanks to the recent availability of the first version of the common bean genome, degradome data and small RNA libraries, we are able to present here a catalog of the microRNAs and phasiRNAs for this organism and, particularly, we suggest new protagonists in the symbiotic nodulation events.

**Results:**

We identified a set of 185 mature miRNAs, including 121 previously unpublished sequences, encoded by 307 precursors and distributed in 98 families. Degradome data allowed us to identify a total of 181 targets for these miRNAs. We reveal two regulatory networks involving conserved miRNAs: those known to play crucial roles in the establishment of nodules, and novel miRNAs present only in common bean, suggesting a specific role for these sequences. In addition, we identified 125 loci that potentially produce phased small RNAs, with 47 of them having all the characteristics of being triggered by a total of 31 miRNAs, including 14 new miRNAs identified in this study.

**Conclusions:**

We provide here a set of new small RNAs that contribute to the broader knowledge of the sRNAome of *Phaseolus vulgaris*. Thanks to the identification of the miRNA targets from degradome analysis and the construction of regulatory networks between the mature microRNAs, we present here the probable functional regulation associated with the sRNAome and, particularly, in N_2_-fixing symbiotic nodules.

**Electronic supplementary material:**

The online version of this article (doi:10.1186/s12864-015-1639-5) contains supplementary material, which is available to authorized users.

## Background

*Phaseolus vulgaris*, known as common bean, is the most important legume for human consumption. This crop is the principal source of protein for hundreds of millions of people and more than 18 million tonnes of dry common bean are produced annually [1]. As a legume, *P. vulgaris* is also a model species for the study of symbiosis in association with nitrogen-fixing bacteria in the genus *Rhizobium*. The recent release of the common bean genome sequence [2] allows the research community to extend their analyses and acquire needed knowledge about this organism. In recent years, a number of studies have focused on gene expression in common bean and its role in a broad range of processes [3] including the response to biotic [4, 5] or abiotic [6, 7] stresses. Gene regulation has particularly been investigated at the post-transcriptional level with the action of small regulating elements called microRNAs (miRNAs) [8–11].

The regulatory processes performed by miRNAs are widely conserved in plants, animals, protists and fungi [12–15], highlighting the significant influence that these regulators can have on the evolution of gene expression. The miRNAs are small non-coding RNA sequences of ~22 nt that negatively regulate gene expression, usually, post-transcriptionally by base-pairing to complementary transcripts. In plants, these small RNAs are processed from longer hairpin-shaped precursors encoded in the genome and almost all of them are transcribed by RNA polymerase II. This pri-miRNA, for primary miRNA transcript, is first processed into a precursor, the pre-miRNA, and then excised as a RNA duplex by the endonuclease enzyme Dicer-like 1 (DCL1). The resulting duplex sequence, composed by the guide miRNA and the complementary miRNA* destined for degradation, is then exported to the cytoplasm by diverse factors, including the HASTY protein. Current knowledge is lacking about the spatio-temporal separation of the two strands but, once performed, the guide miRNA is loaded on to a member of the AGO protein family to assemble the RNA-induced silencing complex (RISC). The miRNA can then play its regulatory role by specifically binding a target transcript based on sequence complementarity. To date, three types of action have been identified for plant miRNAs: cleavage, leading to the degradation of the corresponding transcripts; translational inhibition, disrupting protein production; and in some cases DNA methylation, preventing the transcription of the corresponding genomic locus. *In fine*, the miRNA action leads to the loss of function of the gene by inhibiting protein production (see Voinnet [16] for review).

Plant miRNAs are involved in most, if not all, biological processes and have been found in all the organs where they have been searched for [17]. These sequences are key regulators in important processes such as hormone regulation, nutrient homeostasis, development and interaction with pathogens and symbionts [18–22]. As part of some of these mechanisms and processes, miRNAs can act indirectly on gene regulation *via* triggering the production of other small RNAs. These molecules are called phasiRNAs, for phased small interfering RNAs, including the tasiRNAs (*trans*-acting siRNAs) and other phased small RNAs that require cleavage by miRNAs [23]. Particular transcripts cleaved by microRNAs are recruited by the RNA-dependent RNA polymerase RDR6 and SGS3 to generate double-stranded RNA molecules [24] that are subsequently processed by DCL4 or DCL5 into phasiRNAs of 21 or 24 nt, respectively [25, 26]. Similar to what occurs with miRNAs, these molecules can be loaded on to AGO protein-containing complexes and can direct disruption of protein production by transcript targets, including transcripts distinct from their own production source, but still exhibiting sequence complementarity [27]. Most of the phasiRNAs are produced from protein-coding transcripts and, if not, they are derived from long non-coding mRNAs. Many phasiRNAs target, and are derived from, large protein families such as NB-LRR, MYB and PPR proteins. One recently hypothesized role of phasiRNAs is to target NB-LRR proteins that broadly regulate plant defenses and beneficial microbial interactions [27], which are important features for model legumes such as common bean.

To date, around 10000 miRNAs have been identified in all the Viridiplantae organisms reported in miRBase version 21 [28]. Despite several studies on this topic [29–31], only 8 precursors of miRNAs, generating 10 mature sequences, are referenced for *P. vulgaris* in this database while more than 500 are present for other model legumes such as *Medicago truncatula* or *Glycine max*. The phasiRNAs have not been studied in this organism. Here, we use the recently released genome of common bean [2], 5 small RNA libraries obtained from 5 plant organs, and degradome sequencing to identify a high confidence genome-wide common bean miRNA dataset, the associated target transcripts, and the first *P. vulgaris* phasiRNA catalog ever published.

## Results and discussion

### Overview of the sequencing data

In this study, we used four published high-throughput sequencing libraries of small RNAs [29] obtained from flowers, leaves, seedlings and roots of *Phaseolus vulgaris* and a novel library acquired from symbiotic nodules of the same organism obtained by infection with *Rhizobium tropici*. To identify the whole set of miRNAs and phasiRNAs, we performed this analysis with the recently described genome of *Phaseolus vulgaris* (Phaseolus vulgaris v1.0, DOE-JGI and USDA-NIFA, http://www.phytozome.net/commonbean) [2] as a reference. For the plant organ libraries and the symbiotic nodules, we obtained averages of 3,649,274 and 2,810,685 reads, respectively (Table 1). From these sets, an average of 51 and 37 % of reads were matched to the common bean genome, respectively (Table 1), and 8.3 % of the sequences from the nodule library matched with the corresponding bacterial genome (*Rhizobium tropici CIAT899*, [32]). The lower percentage of reads mapping to the plant genome in the nodule library was partly due to the presence of bacterial sequences and to an increased abundance of ligated adaptor sequences in this particular sample.

**Table 1:** Statistics of the 5 small RNA library genomic mapping

	Filower	Leave	Root	Seedling	Nodule
Raw reads	3356817	3321526	3963174	3955581	2810685
Mapped reads	1756842	1663979	1950627	2138258	1032364
% of mapped reads	52 %	5 %	49 %	54 %	37 %

### Identification and organ distribution of miRNAs

To identify the already referenced and novel miRNAs of *Phaseolus vulgaris*, we used the miRDeep-P pipeline [33] with each library as a set of small RNA candidates and the *P. vulgaris* genome as the corresponding reference. We identified a total of 307 precursors that fulfilled our criteria (see methods) producing 185 unique mature miRNAs (Table 2). 64 of them are already referenced as mature miRNAs in other plant species (miRBase database ver. 21 [28]): these are produced by 111 precursors and distributed throughout 27 families. In this work, we refer to those sequences as “known” miRNAs. Additionally, 57 miRNAs are new isoforms (or family members) [34] of already referenced miRNAs in the miRBase database: these are generated by 98 precursors and distributed in 25 families (Additional file 1: Table S1). We refer to them as “new isomiRs”. Finally, we identified 64 novel miRNAs that are not members of previously described miRNA families. These novel miRNAs are encoded by 98 precursors, grouped in 59 families.

**Table 2:** Statistics of the identified precursors and their corresponding mature miRNAs

	Known	New isomiR	Novel	Total
Identified precursors	111	98	98	307
Identified miRNAs	64	57	64	185
*Relative position*				
Intergenic	97	90	78	265
Intron	1	5	16	22
Exon	11	3	4	18
3’UTR	2	0	0	2
5’UTR	0	0	0	0

In summary, we identified a total of 185 mature miRNAs encoded by 307 precursors and distributed in 98 families. These microRNAs included 64 already known miRNAs, 57 novel isoforms belonging to known miRNA families and a last set of 64 novel miRNAs not identified before. Among the 40 families already registered in miRBase 21 that we have identified (Additional file 1: Table S1), we retrieved all those conserved within the angiosperm genomes, which are the miRNAs from miR156 to miR408 [35]. As expected, the highly conserved miR482, together with the more restricted miR1512 and miR1515, which have been reported as positively regulating nodule number [36], are also present in our dataset. Part of the other identified miRNA families belong to the so-called “legume” miRNAs [37] such as the miR2111, miR2118 and miR2119. Other families retrieved in our libraries had only been identified, up to now, in *G. max* (miR4415, miR4416 and miR5786), *M. truncatula* (miR2597) or both (miR5037). Thanks to the identification of these miRNAs in *P. vulgaris,* we observe that they are now shared between at least two legume species and can properly be called “legume” miRNAs [37]. Interestingly, we also found 5 members of the miR1862 family and 1 member of the miR6175 that so far have only been identified in rice [38] and rubber tree [39], respectively. The members of these two families are sparsely expressed in our libraries and we can imagine that these miRNAs, and others characterized by exhibiting low expression, are highly spatially- or temporally-specific sequences. It is possible that this type of “specific” miRNA is, in fact, present in several organisms and, with the increase in the depth of high-throughput sequencing technologies, will begin to emerge in investigations focused on the identification of miRNAs in less-studied organisms. The miR4376 family may be an example of these. It has been shown to be a super-family derived from the miR390 and was probably present in the common ancestor of Spermatophyta [40], suggesting that this miRNA is conserved in most of the seed plants, but currently only identified in five species: soybean, tomato, ginseng, potato and, now, common bean (miRBase 21).

**Fig. 1:**
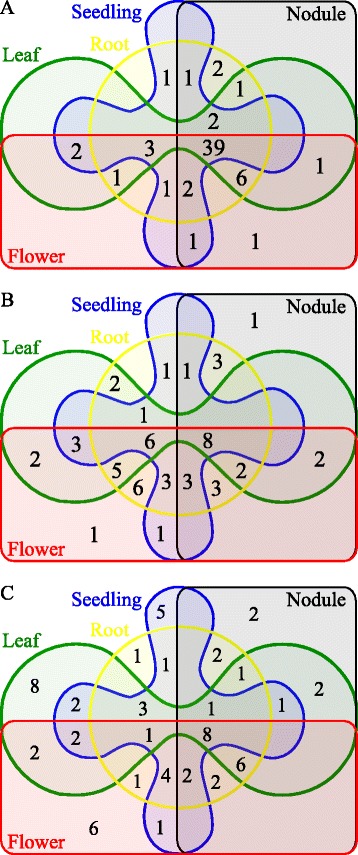
Distribution of the identified mature miRNAs in 5 plant organs. Venn diagram of the distribution of **a** known miRNA, **b** new isomiR and **c** novel miRNA in the different studied organs. Red: flower, green: leaf, yellow: root, blue: seedling and black: nodule. The numbers in each Venn diagram area correspond to the number of mature miRNAs encountered in the corresponding overlapping organ area

By sequencing five small RNA libraries corresponding to five different plant organs, *i.e.*, flower, leaf, nodule, root and seedling, we are able to indicate the distribution of the identified miRNAs in the whole plant based on their expression (see methods). As expected, we found about 61 % of the known miRNAs (39/64) present in all of the organs while only about 14 % and 12 % of the new isomiRs (8/57) and the novel miRNAs (8/64), respectively, are in this class (Fig. 1). In contrast, 22 novel miRNAs are organ-specific whereas none of the known miRNA is. This suggests that the known miRNAs, composed of a majority of conserved miRNAs, play a role at the whole-plant level, as expected from their implication in regulatory networks [41]. In contrast, the newly identified miRNAs, considered as recently emerged and, probably having a more specific action (or no action at all) are characterized by a specific spatial distribution [42]. Alternatively, the characteristic lower expression of isomiRs and novel miRNAs may preclude accurate detection in all organs tested, thus limiting this analysis.

The analysis of miRNA expression and distribution in different organs has been used to identify nodule-specific novel miRNAs in *G. max* and *M. truncatula* [43, 44]. Similar approaches used in this work led us to identify three nodule-specific miRNAs in *P. vulgaris*. These include the new isoform of a conserved miRNA family, the miRNov627 from the mtr-miR399 family and two newly identified miRNAs: miRNov153 and miRNov494. Their putative targets are involved in the regulation of plant-microorganism interactions, which will be discussed below. Other miRNAs with organ-specific expression patterns were detected in our samples and could be also regulating specific processes in other plant tissues (Additional file 2: Table S2).

### Validation of the predicted precursors

**Fig. 2:**
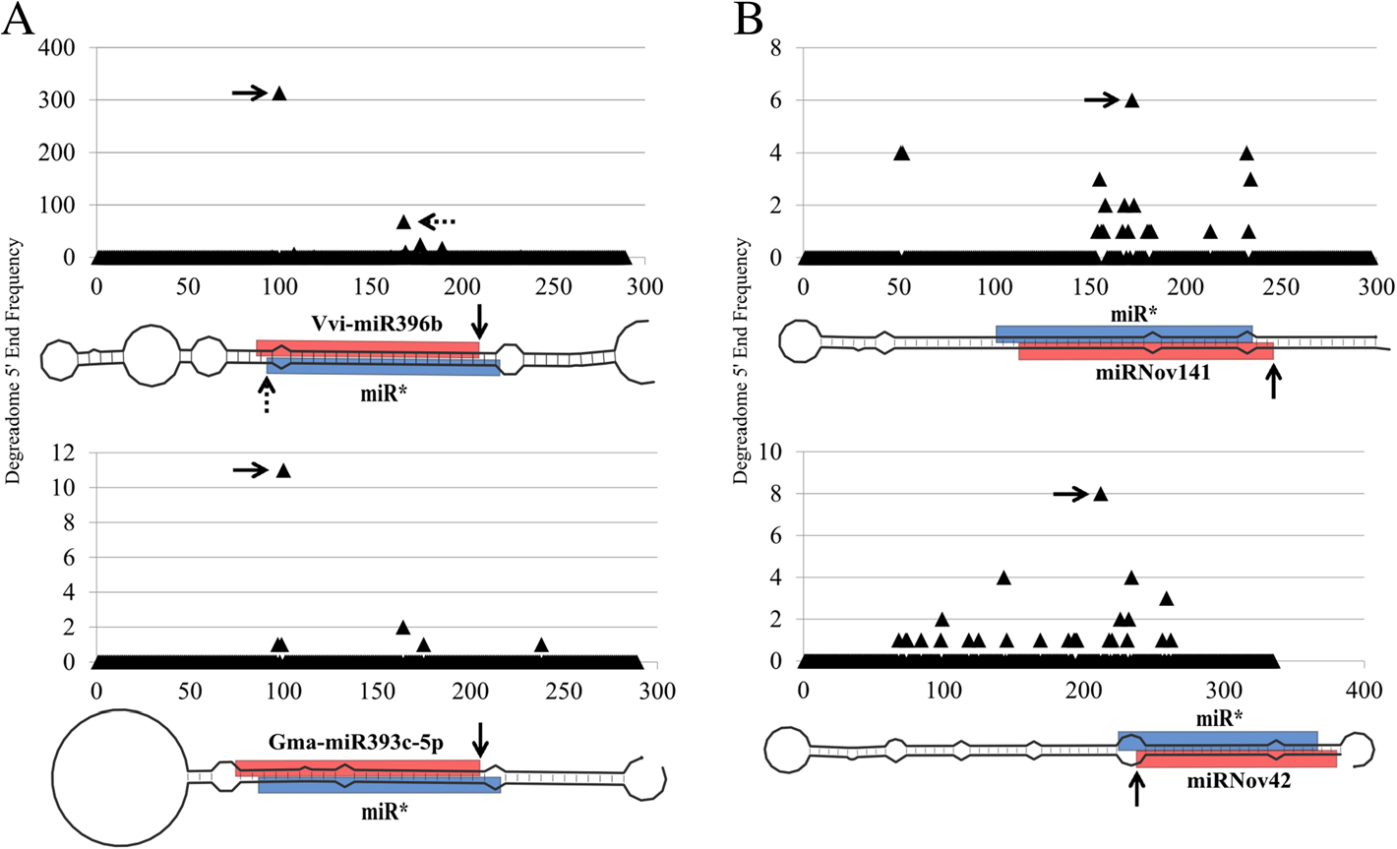
Distribution of degradome signatures on miRNA precursors. Distribution of degradome read abundance on **a** known miRNA and **b** novel miRNA precursors. Vertical axes display the read abundance and the horizontal axes display the precursor position by base pair. The black triangles show the read abundance for the corresponding precursor base. The plain and dotted arrows point to the first base of the mature miRNA and the first base of the miR*, respectively. The schemes below the plots represent the corresponding precursors. The vertical bars show the base pairing and the red and blue boxes represent the mature miRNAs and the miR*, respectively

The recent release of the *P. vulgaris* gene atlas [45] allowed us to verify that the predicted miRNAs have a corresponding transcript in the 24 transcriptome data samples collected from seven distinct tissues of common bean at developmentally important time-points, including plants inoculated with either effective or ineffective *Rhizobium*. We found that about 65, 26 and 24 % of the known miRNA, new isomiR and novel miRNA precursors, respectively, have a complete transcript in at least one of the 24 common bean expression libraries. These validated precursors produce 84, 47 and 35 % of the known, new isomiR and novel mature sequences. To differentiate miRNAs from other small RNAs, we used degradome data to identify the specific signatures of DCL slicing during the miRNA precursor processing. These signatures are characterized by the finding of a significant processing event at the mature miRNA and/or miRNA* boundaries within the precursor [46]. With this method, we validated the correct processing of 41 % of the known miRNA precursors, 30 % of the new isomiR precursors and 22 % of the novel miRNA precursors, thus constituting the validation of 63 % of the known mature miRNAs, 44 % of the new mature isomiRs and 33 % of the novel mature miRNAs (Fig. 2). The failure to validate some miRNA precursors could be due to undesirable RNA degradation producing random degradome signatures, or the presence of degradome signatures originating from other loci that mask the expected significant signatures [47]. It is also possible that some precursors are not functional and mature miRNAs only originate from a subset of potential precursors within the same MIR family. Compared with the precursors encoding well-conserved miRNAs, a lower proportion of precursors for novel miRNAs allowed degradome validation. A general problem encountered with these sequences is their lower expression levels and corresponding lower degradome signatures, the latter being obscured by other gene degradation products.

Finally, if we combine the results of the expression data with those of the degradome analysis, we obtain supporting data for 78, 47 and 35 % of the known, new isomiRs and novel miRNA precursors we encountered, respectively, representing 92, 54 and 44 % of the corresponding mature miRNAs originally identified. Overall, 64 % of the identified mature microRNAs show evidence of expression for at least one of their proposed precursors by at least one of the two methods examined.

### miRNA identification aided by genomic sequence data

The present study profited from the use of the recently released complete genomic sequence for *P. vulgaris* [2] to identify miRNAs. Compared to the previous study of Peláez *et al.* [29], this new approach allowed us to confirm the presence of 81 of the 113 known miRNAs identified by Peláez *et al.* in *P. vulgaris*. 25 out of the 32 miRNAs previously identified by Peláez *et al.* and not encountered in our study are actually not present in the current version of the genome and the remaining seven were more precisely defined in our study. Four of the seven non-identified miRNAs have been detected in the libraries but we designated other more abundant isoforms or novel miRNAs as mature miRNAs in the corresponding precursors. The 3 other miRNAs were not identified by miRDeep-P because of potential splicing events in the hairpin sequence or because of a non-conventional folding of the stem-loop. In contrast, our study revealed 4 already identified mature miRNAs in the miRBase catalog of other species and 34 new isomiRs in addition to the set proposed by Peláez *et al.* Concerning the novel miRNAs, none of those identified by Peláez *et al.* fulfilled our selection criteria. We identified only one isomiR of a novel miRNA previously described by Peláez *et al.*, 21 could not be mapped to the genome and 17 were present in more than 40 locations in the genome and thus were not further considered (see methods). The other 19 miRNAs were not selected because they do not fulfill our folding, splicing or expression criteria. Conversely, the availability of genomic sequence data allowed us to identify 64 high-confidence novel miRNAs that satisfy all our criteria and all those currently accepted for microRNA annotation [48]. Means of 2.5 and 6.9 hits in genome for each known and novel miRNA were found, respectively. We can ascribe this difference to a lower selection pressure on the novel miRNAs [49], compared to conserved ones, and the fact that most of these newly identified miRNAs are young miRNAs and, perhaps, their selection process is still occurring.

In summary, the new whole-genome investigation of the miRNA candidates allowed us to identify 307 genuine precursors of already known and new miRNAs and 121 high confidence unpublished mature sequences.

### MiRNA precursor genomic localization

In plants, most of the miRNA genes are encoded in intergenic regions [16, 50] but some are present in introns, exons, or UTRs [51]. In our study, we localized the different precursors of miRNAs in the genome and determined their position relative to annotated genes. As expected, about 90 % of the known and new isomiR are located in intergenic regions (Table 2). One of the known miRNAs, pvu-miR1514a, has a precursor encoded in an intron and two, bna-miR167d and gma-miR167a, are located in 3’UTRs. There are 11 other known miRNAs located in exons. The proportion of known miRNAs located in exons of annotated genes is high compared to that of miRNAs present in other gene locations; however, due to the lack of curation in the current annotation of the *P. vulgaris* genome this could be an overestimation. These sequences are located in exons of putative small proteins lacking known domains or homologs in other organisms and it is very likely that these precursors are, in fact, intergenic. For the novel miRNAs compared to known ones and new isomiRs, we encountered fewer precursors in intergenic regions (~80 %, Table 2) but more in introns (~16 %). Since we found a higher proportion of novel miRNAs in introns, we can imagine that some of the younger miRNA genes could originate from introns. There are different hypotheses on the origin of the miRNAs: novel miRNA genes may originate from duplication of other miRNAs, from inverted terminal repeats of transposable elements or from random stem-loop structures emerging from intergenic or intronic regions (see Zhuo *et al.* for review [50]). In this sense, the birth of a miRNA gene is less costly if it derives from a ready-to-use transcription unit that an intron can confer indirectly [52]. In our results, 15 of the 16 precursors of novel miRNAs identified in introns are located in the same orientation as the corresponding gene, supporting this hypothesis. On the one hand, the expression of various miRNAs located in introns is dependent on the expression of the corresponding host gene [53] and we can think that the evolutionary selection of this type of transcription mechanism is fixed. On the other hand, we can hypothesize that some of the miRNAs present in introns are young miRNAs and the use of the transcriptional unit of the host gene is a first step in the evolution and, thereafter, they may acquire their own transcriptional unit and evolve as canonical independent miRNAs thanks to duplication events or exon shuffling [54].

### Conservation of the identified miRNAs in plants

**Fig. 3:**
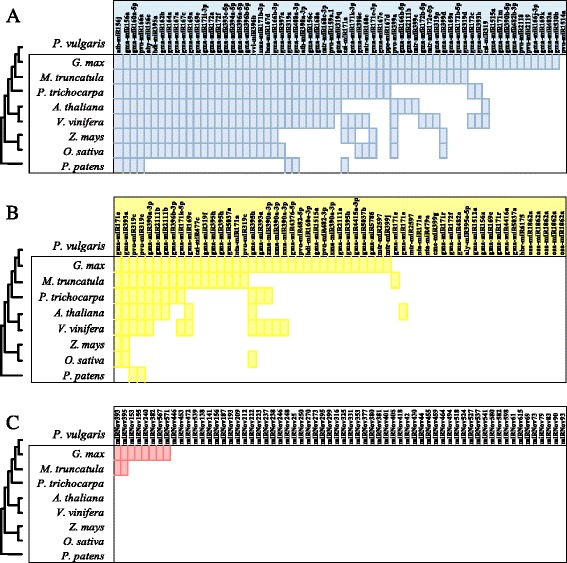
Conservation of the *Phaseolus vulgaris* mature miRNAs in 8 plant species. The different mature miRNAs studied are listed on the first row of the tables. The first column indicates the organisms where the sequences have been searched for and the other columns correspond to the different **a** known miRNAs, **b** new isomiRs and **c** novel miRNAs. The presence of a colored square at the intersections between rows and columns indicates the 100 %-homology presence of the corresponding mature sequence in the genome of the corresponding species. The tree on the left indicates the phylogenetic relationship between the studied species

We investigated the conservation of the identified miRNAs in 7 vascular plants and 1 moss. We chose two legumes (*Medicago truncatula* and *Glycine max*), three non-legume eudicots (*Vitis vinifera*, *Populus trichocarpa* and *Arabidopsis thaliana*), two monocots (*Oryza sativa* and *Zea mays*) and the moss *Physcomitrella patens*. A miRNA is considered as conserved in a given species when it is present in its full length without any mismatches. As expected, in a global view, the known miRNAs are the most conserved with 4 miRNAs (ath-miR156j, gma-miR156a, gma-miR160a and pvu-miR166a) present in all the selected genomes. We found 21 miRNAs, including 19 known and 2 new isomiRs (pvu-miR319c), conserved in all the plants except the moss (Fig. 3). A total of 118 miRNAs seem to be legume-specific: 22 % of the known miRNAs, 73 % of the new isomiRs and 100 % of the novel miRNAs. Among them, 77 are specific to *P. vulgaris*: 1 known miRNA (pvu-miR1514a), 20 new isomiRs and 56 novel miRNAs.

The proportion of species-specific miRNAs is variable in plants. For example, in *Arabidopsis thaliana* and *A. lyrata*, 35 % of the identified miRNAs are species-specific [55]. This is also the case of 23 % of the *Medicago truncatula* miRNAs [44], 37 % in *Populus* species [56], 41 % in wheat [57] and 56 % in apple [58]. In our results we found that *P. vulgaris* is in the range of these other plants with around 42 % of the miRNAs specific to the common bean. Naturally, these numbers may fluctuate depending on the depth of sequence obtained and the methods used for the identification and the prediction of the microRNAs. The lack of conservation for the specific miRNAs suggests that they emerged recently during evolution and can thus be considered as young miRNAs.

### Prediction and identification of miRNAs targets

**Fig. 4:**
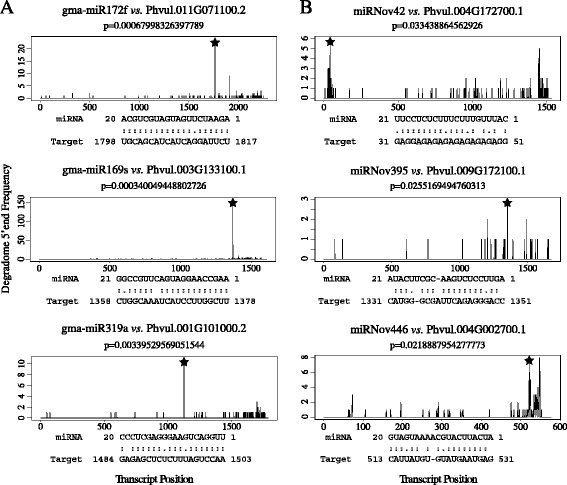
Target plots of miRNA-targeted transcripts using degradome data. Target plots of the distribution of degradome read abundance on transcripts targeted by **a**. known miRNAs and **b**. novel miRNAs. Vertical axes display the read abundance and the horizontal axes display the precursor position by base pair. The black vertical bars show the read abundance for the corresponding precursor base. The black stars indicate signatures consistent with the miRNA-directed cleavage. The corresponding *p*-values are shown above the plots

**Fig. 5:**
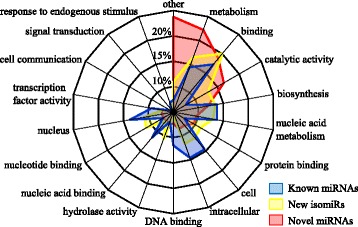
Distribution of the degradome target function. Radar plot of the distribution of the degradome target functions. Each branch corresponds to a different gene ontology category. The proportion (%) of known miRNA (*blue*), new isomiR (*yellow*) and novel miRNA (*red*) degradome targets that belong to each functional category are indicated by the distance from the center of the colored area intersection with the corresponding branch. The “other” category represents all the functional categories displaying less than 2 % of the targets

Using degradome data and the prediction of putative targets, we are able to present a set of targets for the identified miRNAs. The degradome data reported here were obtained using a method based on the 5'-rapid amplification of cDNA ends (5’RACE) and further adapted for high-throughput sequencing. It allows the experimental identification of the target cleavage sites associated with miRNA cleavage on a genomic scale [59]. The regulation performed by miRNAs does not necessary include a cleavage of the target transcript. To complete the degradome data, we used a target prediction tool, called psRNATarget [60], based on reverse complementary matching between miRNA and target transcript. A mean number of one degradome target and 6.5 predicted targets per miRNA candidate were found (Additional file 3: Table S3). The known miRNAs possess significantly more degradome targets (1.9/miRNA) compared to the novel ones (0.3/miRNA) and also have more predicted targets (8.8/known miRNA *vs.* 5.8/novel miRNA). Several of the target transcripts identified here for known miRNAs correspond to previously defined targets found in other plant species, thus validating our analysis. As discussed in previous studies, the lack of conventional targets for the young miRNAs is not unusual. Most of the young miRNAs, in contrast to the conserved ones, are not involved in complex regulatory networks [41] and their evolution has sometimes been considered as neutral [49]. However, the experimental demonstration of an interaction with its predicted target is considered as “the most powerful method of validating a predicted miRNA” [61]. Although 73 % of the conserved miRNAs exhibit a degradome target in our data, only around 28 % of the novel miRNAs also have a target identified by degradome analysis (Fig. 4). Here we found that a significant portion of novel miRNAs has functional evidence, as revealed by degradome data, for a role in regulating gene expression, thus suggesting their relevance in different biological processes. Their degradome targets are for the most part involved in metabolic processes, biosynthesis, binding processes and various functions (Fig. 5). Compared to the conserved miRNAs that preferentially target genes involved in complex regulatory networks such as transcription factors [62], young miRNAs tend to target more precise and diversified functions. However, one of them, miRNov138, seems to target a transcript coding for a Homeobox-leucine zipper family protein. This transcription factor family is known to play an important role during the growth and development of plants by modulating phytohormone-signaling networks [63] and also in the interaction with microorganisms, especially during nodulation [64]. This young miRNA is specific to *P. vulgaris* and we hypothesize that it has been selected to perform a more precise role in common bean, like adaptation to a specific environment [63] or interaction with *Rhizobium*.

### Analysis of microRNA co-expression networks in nodules

To understand the role of the newly identified miRNAs in nodules, we constructed weighted correlation networks of miRNAs. These networks describe the pairwise relationship among miRNAs that differentiate the nodule library from other libraries based on the miRNA expression patterns [65]. Using this approach, we identified 2 networks including pairwise relationships between novel miRNAs, new isomiRs and conserved miRNAs.

**Fig. 6:**
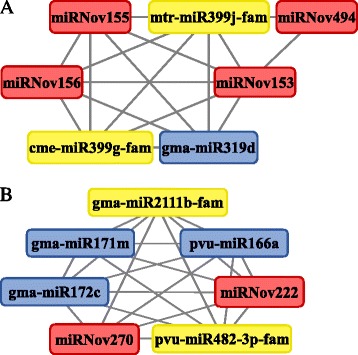
Weighted correlation network analysis of microRNAs from nodules versus other organs. Representation of two regulatory networks **a **& **b** between the miRNAs. The nodes correspond to each miRNA encountered in the corresponding network. The edges correspond to the direct connection and relation between the nodes. *Blue*, *yellow* and red tags indicate the known miRNAs, new isomiRs and novel miRNAs, respectively

**Fig. 7:**
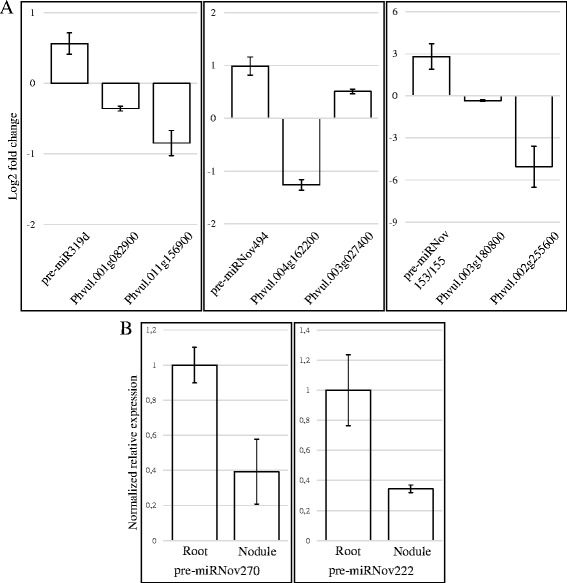
Expression quantification of miRNA precursors and their targets involved in the weighted correlation networks. Relative quantification by qRT-PCR of miRNA precursors and their corresponding targets. **a** Expression of miRNAs and corresponding targets in nodules compared to root tissues from the Fig. 6A network. Histograms represent the Log2 fold change level and error bars represent the standard error of the mean of the three expression ratios from three biological replicates. **b** Expression of novel miRNA precursors from Fig. 6B network. Histograms represent the relative expression and error bars represent the standard error of the mean of the three relative expressions from three biological replicates. Expression values have been standardized at 1 for the control roots and proportionally adjusted for nodule expression. The geometric mean of the expression of three housekeeping genes was used for normalization of the expression levels

One of these networks is composed by 4 novel miRNAs (miRNov153, miRNov155, miR156 and miRNov494), two new members of the miR399 family, and gma-miR319d suggesting that they could act jointly (Fig. 6A). All these miRNAs have the same expression pattern, with increased expression in nodules (Additional file 1: Table S1). As shown in Fig. 7A, increased expression of pre-miR319d, pre-miRNov153/155 and pre-miRNov494 in nodules was experimentally validated by qRT-PCR. More precisely, the new isoform of the mtr-miR399j was specifically identified in nodule (Additional file 2: Table S2). This miRNA family is involved in phosphate homeostasis [66] but, furthermore, has been identified as being repressed by N-starvation [67]. Nodulation efficiency and functionality are related to these two nutrients [68, 69] and we hypothesize that this new isoform of miR399 could indirectly regulate the normal establishment of nodulation in *P. vulgaris*. Indeed, this miRNA family is known to target a transcript encoding PHO2, an ubiquitin-conjugating enzyme crucial for acquisition and translocation of phosphate [70]. In our data, we predicted another target for the new isoform of miR399 which could regulate a NB-ARC domain-containing protein, one of several factors known to be involved in the plant resistance and activation of innate immune responses [71]. The organ-specific accumulation of this miRNA and the function of the corresponding target suggest a role in the regulation of nodule-specific defense mechanisms. Two of the novel miRNAs from this network; miRNov153 and miRNov494, are specific to the nodule library. The miRNov494 is predicted to target two transcripts in our degradome data (Additional file 3: Table S3). qRT-PCR expression analysis showed that in nodules the expression of miRNov494 precursor increased while the expression of one of the predicted targets, an aldehyde dehydrogenase (Phvul.004G162200.1) decreased (Fig. 7A). The members of this protein family are known to change their expression in response to a wide variety of stresses and are important in supporting environmental adaptability [72]. Our data suggest that the miRNov494 can regulate a member of the aldehyde dehydrogenase family in nodules and may help the plant to control the life cycle of this symbiotic organ. MiRNov153 is ~18 times more highly expressed than the miRNov494 and is encoded in an intron of an F-box protein gene (Additional file 1: Table S1). Part of this protein family plays important role in root symbioses [73] and particularly in the auto-regulation of nodulation [74]. This novel miRNA, miRNov153, is predicted to target an uridine kinase (Phvul.003 g180800), for which no expression variation has been measured in nodule compared to root tissue, and a transcript coding for a hypothetical protein (Phvul.002 g255600), for which we have observed an expression inversely correlated to its regulator miRNA in nodule (Fig. 7A). Although this target does not guide us to an obvious functionality, the fact that this miRNA is encoded in an intron of an F-box protein gene, in the same orientation, would permit us to envisage that this miRNA is co-expressed with the host gene and could act in the regulation of the systemic negative feedback control of nodulation. Among the 8 species for which we searched for the presence of the miRNov153, only the soybean genome encodes this miRNA and displays a *bona fide* hairpin-like precursor (Additional file 4: Figure S1A). To check if this locus can produce a miRNA corresponding to the mature miRNov153 in soybean, we mapped public nodule small RNA sequences from Arikit *et al.* [75] to the miRNov153 soybean precursor (Additional file 4: Figure S1B). Tens of sequences mapped exactly to miRNov153 and the corresponding miRNov153* and only three sequences mapped to other coordinates, suggesting that nodules from soybean also produce the miRNov153. The available public small RNA libraries from nodule represent 5 time points from 10 dpi to 30 dpi. In these libraries, we observe an increased expression of the miRNov153 during nodule development. Additionally, this miRNA is absent from *Medicago truncatula,* which develops indeterminate nodules, and is conserved in common bean and soybean, two species developing determinate nodules. These results tend to show a role of miRNov153, *via* its targets, in the loss of meristematic activity or the senescence of determinate nodules.

To our knowledge, no link has been published before between miR319 and nodulation. This miRNA is a regulator of the plant stress responses against salinity, drought or cold [76, 77] but it is also implicated in the regulation of cell proliferation *via* the control of its targets, members of the TCP transcription factor family [78]. Here, we have validated the increase of miR319d precursor expression in nodules and the decrease of one of its predicted targets, a TCP transcription factor family member (Phvul.011 g156900), in the same tissue (Fig. 7A). Because this miRNA is present in a network that distinguishes the nodule library from the others, and because it is connected with miRNAs described in previous studies as being related to nodules, we hypothesize that miR319 and its targets are strong candidates for which a role in nodule development must be investigated.

We identified a second network involving 7 miRNAs: 2 novel ones, 2 new isomiRs and 3 already known miRNAs (Fig. 6B). In this network, we retrieved members of miRNA families already described as regulating nodule development. Specific variants of *L. japonicus* and *M. truncatula* miR171 target the GRAS-family *NSP2* TF, a key regulator of the common symbiotic pathway for rhizobial and arbuscular mychorrizal symbioses [79–81]. MiR166 and its target gene, a HD-ZIP III transcription factor, regulate meristem activity and vascular differentiation in roots and nodules [64]. In soybean, the over-expression of miR482, targeting a GSK-3-like protein MsK4, resulted in an increase in nodule number without affecting root development or the number of nodule primordia [36]. The crucial role of miR172 and its target gene, a transcription factor from the AP2 family, have been described for soybean- and common bean-rhizobia symbiosis [82–84]. For soybean, Yan *et al.* [82] postulated that miR172 regulation of nodulation is explained by the *AP2* repression of non-symbiotic hemoglobin (*Hb*) gene expression that regulates the level of nodulation; while Wang *et al.* [84] recently reported that soybean NNC1, a AP2 transcription factor target of miR172, represses *ENOD40* expression that results in negative regulation of early symbiotic stages. For common bean, we recently demonstrated [83] that miR172c, which has the transcription factor AP2-1 as target gene, indirectly regulates the expression of the transcription factors NF-YA1, NSP2 and CYCLOPS as well as the gene *FLOT2*, all of which are essential regulators of early stages of the symbiosis. In addition, we postulated that miR172-induced AP2-1 silencing in mature common bean nodules is involved in down-regulating expression of genes related to nodule senescence postulated as targets of AP2-1 transcription activation [83]. We also found a new isomiR of a family already described as regulating the development of nodules: the miR2111 targeting a Kelch-related proteins [85]. Finally, the last two miRNAs encountered in this network are new miRNAs: miRNov222 and miRNov270. Although these miRNAs have low expression and present no processing evidences in degradome data or transcription evidences in transcriptome data (Additional file 1: Table S1), we have detected the corresponding precursors by qRT-PCR in roots and confirmed their expression decrease in nodules (Fig. 7B). As elements in a network containing miRNAs previously described as nodulation regulators, these novel miRNAs are also probably involved in the normal establishment and functioning of the nodulation process *via* regulation of their targets. These two miRNAs putatively target a protein kinase and a NB-ARC domain-containing disease resistance protein, respectively (Additional file 3: Table S3). Although the protein kinases belong to a large protein family, various members are known to be involved in signalling during nodulation [86] and, as described above for the putative target of the new isoform of the miR399, the NB-ARC domain-containing disease resistance proteins are known to be involved in the plant resistance and activation of innate immune responses [71] and could play a specific role in the nodule and allow the proper functioning of the nitrogen-fixing organ. These novel miRNAs are directly related to regulators of nodulation and it is conceivable that these *Phaseolus*-specific miRNAs could act in the same way as the connected conserved miRNAs and play a role during nodule establishment in a more spatio-temporal specific manner. Like the miR319 family, these novel miRNAs must be considered as major candidates for deciphering the specific regulation of nodulation in *P. vulgaris*.

### Identification of phasi-RNAs and their associated targets

**Fig. 8:**
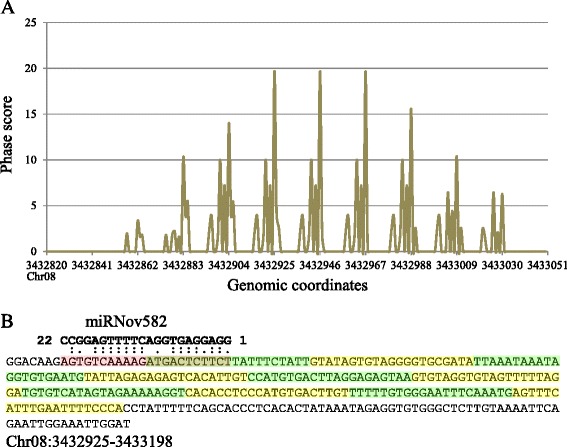
Identification of a phasiRNA loci and their triggering miRNAs. **a** Distribution of the phase score (vertical axis) as a function of the genomic coordinates (horizontal axis) of the *PHAS* gene Chr08:3432925–3433198 coding for a pfkB-like carbohydrate kinase family protein. **b** Representation of the miRNov582 triggering the 21 nt phasiRNA production (green and yellow alternation) of the *PHAS* gene Chr08:3432925–3433198. The G–U hydrogen bonds are represented by a single dot and the A–U and G–C hydrogen bonds by two dots

In all the sequenced libraries, a large number of reads do not correspond to miRNAs or other previously identified non-coding RNAs of common bean, suggesting they could belong to another class of small RNAs. We decided to focus on phasiRNAs and, based on the alignment of the reads with the genome and the phasing score for each locus, we predicted 125 statistically significant loci producing 21 nt-phased small RNAs (Additional file 5: Table S4, see example in Fig. 8A). Among the identified *PHAS* genes, only two are conserved in the three legumes studied, *i.e. M. truncatula*, *G. max* and *P. vulgaris* (Additional file 6: Figure S2). One of these *PHAS* genes is known as the *TAS3* gene targeted by the miR390 and producing small RNAs targeting transcripts that encode AUXIN RESPONSE FACTORs (ARF3 and ARF4) [87]. In our data, we also identified miR390 and its regulatory action on the *TAS3* gene (Additional file 5: Table S4), providing a positive control for our methodology. The second *PHAS* gene codes for a member of the disease resistance protein (TIR-NBS-LRR class) family known to be targeted by miR2118 in soybean but not yet in *M. truncatula* [71]. In our analysis, we encountered that miR2118 potentially targets this *PHAS* gene but not in the expected phase. However, for this gene, we have identified a potential in-phase slicing by a novel miRNA, the miRNov212 (Additional file 5: Table S4). Among the 125 loci identified, 47 are predicted to be targeted by 31 different miRNAs with a cleavage site in phase with the initiation site of the corresponding phasiRNAs. In recent years, loci producing 21 nt-phasiRNAs (*PHAS* genes) have been identified in different species and their number in each case varies widely: 50 in *Arabidopsis*, 114 in *M. truncatula,* 157 in apple, 353 in peach and 864 loci in rice [40, 71, 88–90]. Not all *PHAS* genes are associated to a miRNA triggering phased small RNA, and the proportion of *PHAS* genes targeted by a miRNA also varies from species to species. For example, 26 of the 157 apple *PHAS* genes and 160 of the 864 rice *PHAS* genes are potentially targeted by miRNAs that can trigger phasiRNA production. Here, we identified 13 known miRNAs, 4 new isomiRs and 14 novel miRNAs that potentially trigger the production of phasiRNAs (an example is shown in Fig. 8B). Most of the 17 previously identified miRNAs are known to be involved in the production of phasiRNAs in other species [27, 91]. The phasiRNAs produced by these known miRNAs originate from transcripts encoding proteins involved in a wide range of functions like the MYB genes targeted by miR159, NB-LRR genes targeted by miR482 and miR2118, Ca^2+^-ATPases targeted by miR4376 or TIR/AFB targeted by miR393 [27]. Uncharacteristically, we identified one *PHAS* gene (Chr11: 2402498–2402857) that possesses all the features to produce two sets of phasiRNAs derived from two distinct phases that are triggered by two novel miRNAs (miRNov141 and miRNov316). The resulting phasiRNAs are only expressed in the flower and the corresponding miRNAs are almost uniquely expressed in the same organ (Additional file 2: Table S2). These phasiRNAs are produced from a transcript coding for a putative serine carboxypeptidase-like 35, a member of a large family involved in multiple cellular processes. In rice, a member of this family has been identified as playing a role in defense against pathogens and oxidative stress [92]. The mechanism of double-phasing by two different miRNAs allows production of two overlapping sets of phasiRNAs from the same sequence and we can imagine this confers an additional layer of small RNA production allowing the more precise regulation of defense reactions of the organism. The complete set of *PHAS* genes targeted by microRNAs must be larger than the number reported here, since we may not have a complete list of miRNAs yet. In addition, other miRNAs may generate phasiRNAs by cleaving target transcripts at an out-of-phase position by a phenomenon called phase-drift, caused by a DCL slippage [93], and, identification of these cleavage events becomes challenging.

All of the studied organs presented transcripts producing 21-nt phasiRNAs but only 13 of the 125 *PHAS* genes (~10 %) were identified in the five different small RNA libraries (Additional file 7: Figure S3). Moreover, most of the phasiRNAs are organ-specific (~73 %) and flowers present the largest set of identified phasiRNAs expressed, with around 73 % of the loci, including ~69 % of organ-specific expressed loci. Conversely, nodules had lower expressed phasiRNAs, with ~13 % of the identified loci. 86 of the 125 *PHAS* loci are localized in a predicted transcript, including 46 with an associated putative function (Additional file 5: Table S4) suggesting that the corresponding phasiRNAs can also target these transcripts or other members of the corresponding gene families. Among the phasiRNA loci localized in transcripts with a putative function, two are expressed in all the organs and are derived from an Auxin signaling F-box 2 and a NAC transcription factor-like 9. These two proteins are related to auxin signaling and lateral root formation [94] and the corresponding families are known to be common targets of phasiRNAs [40]. The presence of the corresponding phasiRNAs in all the studied organs reflects the important role in regulation of these small RNAs at the organism level. MiR2118 is predicted to target two *PHAS* genes (Chr04:3128306–3129229 and Chr04:9380782–9381299) coding for proteins involved in disease resistance: a NB-ARC domain-containing disease resistance protein and a Disease resistance protein (TIR-NBS-LRR class) family member. These loci are expressed in all the organs except nodules (Additional file 5: Table S4). Although miR2118 has been retrieved in all the organs studied, including nodules, we can hypothesize that miR2118 regulates nodule growth *via* the control of phasiRNA production derived from the NB-ARC and TIR-NBS-LRR transcripts. The investigation of phasiRNAs is quite recent and we envisage that, in coming years, the number of *PHAS* genes will increase, as it has for miRNAs in recent years, in terms of loci per species and number of studied plant species.

## Conclusions

Thanks to the genome-wide identification of miRNAs and phasiRNAs from *P. vulgaris*, we provide a set of sequences allowing the extension of the sRNAome of common bean. The investigation of the miRNA degradome targets, the miRNA correlation networks and the *PHAS* gene function permitted us to propose a role for the identified small RNAs and provide genuine sequence candidates to be studied in plant-microorganism interactions and specifically in the root-nodule symbiosis.

## Methods

### Plant materials and sequencing

#### Plant materials

Flower, leaf, root and seedling raw sequences were obtained from the study of Peláez *et al.* [29]. Briefly, *Phaseolus vulgaris* L. cv. Negro Jamapa and cv. Pinto Villa were grown and roots (15 day old) and flower buds (35–40 day old) were collected in liquid N_2_ and stored at - 80 °C. Whole 1–4 days old seedlings were collected in liquid N_2_ and stored at −80 °C. A pool of leaves from 10 and 20 day-old plants was harvested for RNA purification.

#### Nodulation conditions

*P. vulgaris* L. cv. Negro Jamapa seeds were surface sterilized and germinated under sterile conditions for two days and then planted in pots with sterile vermiculite. A fresh inoculum of *R. tropici* CIAT899 grown on PY liquid medium supplemented with CaCl_2_ (7 μM), rifampicin (50 μg/ml), and nalidixic acid (20 μg/ml) at 30 °C to a cell density of 5 × 10^8^ ml^−1^ was prepared. Immediately after transferring bean plants into pots with fresh sterile vermiculite, each plant was inoculated by adding 1 ml of the *R. tropici* culture directly to the root. Plants were grown in a greenhouse with a controlled environment (25–28 °C, 16 h light/8 h dark) and were watered with nitrogen-free B&D nutrient solution [95] every 2 days. Root nodules were collected 18 and 27 days after inoculation.

#### Library preparation and sequencing

Total RNA was isolated from frozen samples using the Trizol reagent according to the manufacturer's instructions (Invitrogen, Carlsbad, CA) and 10 μg of each sample was prepared for Deep Sequencing following Illumina's Small RNA alternative sample preparation protocol v1.5. Complementary DNA (cDNA) libraries were separately prepared and Single Read-sequenced using the Genome Analyzer IIx (GAIIx) (36 bp) and the Illumina Cluster Station (Illumina Inc, USA) at the Instituto de Biotecnología (Universidad Nacional Autónoma de México) and raw reads from the *Illumina Pipeline 1.4* for the small RNA libraries were purged of sequence adapters, low quality tags and small sequences (<16 nt long) [29]. The contamination of the nodule library by bacterial sequences was investigated by BLASTn of the whole nodule small RNA set against the *R. tropici* CIAT899 genome as a reference. Raw reads for the small RNA sequencing are available in the Gene Expression Omnibus database under accession number GSE67409.

### Degradome library construction

#### Plant material

Seeds of *P. vulgaris*, var. Pinto Villa were surface sterilized in 50 % (v/v) sodium hypochlorite and 0.5 % (v/v) Tween-20 for 3 min, rinsed with distilled water for 10 min and germinated in wet paper towels in the dark at 24 °C for 3 days. At this point seedlings of similar size were transferred to moist vermiculite watered to substrate capacity and maintained at 24 °C for 48 h with 16 h/8 h light/dark day cycles. Seedlings were collected, ground to a fine powder under liquid N_2_ and preserved at −80 °C until the material was used for RNA preparation.

#### Library construction for high throughput sequencing

The protocol used to obtain cDNA libraries for degradome analysis was carried out essentially as previously described [59, 96]. Total RNA from seedlings was obtained using the Trizol reagent (Life Technologies) according to the manufacturer’s directions. Subsequently, poly-A+ RNA was ligated to a 5’adapter containing a MmeI site at its 3’-end. The ligated products were used for cDNA production and amplified by PCR for five cycles. The PCR products were digested with MmeI and the resulting fragments ligated to a second double-stranded oligonucleotide, purified and amplified for another ten PCR cycles. The final product was purified and subjected to high throughput sequencing as described below.

#### High Throughput sequencing

DNA libraries were subjected to Single Read-sequencing (36 bp) using a Genome Analyzer IIx (GAIIx) and the Illumina Cluster Station (Illumina Inc, USA) at the UNAM Sequencing facility (Unidad Universitaria de Secuenciación Masiva de DNA, Universidad Nacional Autónoma de México). Raw reads for the degradome sequencing are available in the Gene Expression Omnibus database under accession number GSE67432.

### Bioinformatics analysis of sequencing data

#### miRNA identification

The identification of the miRNA precursors was performed using the miRDeep-P pipeline [33] with a maximal identification window size of 250 nt, no mismatch allowed and a number of hits in the genome lower than 40. The precursors with a small RNA overlapping an ncRNA (tRNAs, rRNAs and other ncRNAs) were discarded by the software. We recovered only the precursors with a size of mature miRNA between 18 nt and 25 nt wherein the mature miRNA lies within the top 5 % of the most expressed sequences, in a given library. The selected mature miRNAs were compared with all the Viridiplantae miRNAs of the miRBase version 21 [28] using the NCBI BLASTn program [97], allowing no mismatches to identify the already known miRNAs. To identify the miRNAs families and the new isoforms of already known miRNAs, we used CD-HIT (Cluster Database at High Identity with Tolerance) [98] with at least 84.2 % of identity. For the novel miRNAs, a more stringent selection was performed and only those mature sequences for which the best precursor miRDeep score is greater than 2.2 (the threshold for the top 5 % of the precursor miRDeep scores) have been kept. To list all the precursors encoding for the final set of novel genuine mature miRNAs, we recovered all the corresponding precursors that have been identified by miRDeep-P and passed all our filters without a selection on the attributed score.

#### Target identification

Two programs were used to identify the targets of the selected miRNAs. CleaveLand ver.4 [99] was used to identify the putative target sites from degradome data (see Degradome library construction section). The transcripts from the version 1 of the *P. vulgaris* genome [2] were used as target templates, the total set of identified miRNAs has been used as the small RNA candidates and only targets with a p-value lower or equal to 0.05 were selected. psRNATarget [60] was used to predict putative miRNA targets on the same transcript dataset. Default parameters were used and only the targets with an expectation value lower than 3 were retained.

To identify the functional distribution of these sets of targets, we used the corresponding Gene Ontology Annotation [100] and classified the corresponding GO terms with CateGOrizer [101] using the GO_slim2 classification with the “accumulative all occurrences” count method.

#### Organ distribution and conservation analyses

The organ distribution was performed manually: a miRNA is considered as present in an organ when the corresponding mature sequence is in the top 5 % of the most expressed mapped sequences of the corresponding organ library. The “five sets” Venn diagrams were inspired by Edwards’ Venn diagrams [102].

Conservation of mature miRNAs was investigated using the NCBI BLASTn program [97] against the genomes of 8 species: two Fabaceae (*Medicago truncatula* 4.0 and *Glycine max* Wm82.a2.v1), three non-legume eudicots (*Vitis vinifera* Genoscope.12X, *Populus trichocarpa* 3.0 and *Arabidopsis thaliana* TAIR10), two monocots (*Oryza sativa* 7.0 and *Zea mays* 6a) and the moss *Physcomitrella patens* 3.0. No mismatches were allowed in identifying the corresponding homologous sequences.

#### Weighted correlation network analysis of microRNAs

The weighted correlation networks were constructed with the WGCNA package for R [65, 103] following the automated one-step protocol and with the default parameters except the minimum module size of 3 miRNAs in order to discover the smallest networks. We used normalized expression data of the 185 mature miRNAs according to the formula: (miRNA read number * 1.000.000)/total mapped reads per library. The eigengene value was calculated for each module and networks in order to test the significant association with the nodulation trait (the difference between the nodule library and the other libraries). Then, the networks were drawn using Cytoscape [104].

#### PhasiRNA identification

Bowtie alignments [105] without mismatch were used to predict the phasiRNAs. Phasing score [71] was calculated for each 21 nt read mapped to the genome with a threshold of 15 as mentioned in Zhai *et al.*, [71]. To eliminate the contribution of random fragments generated from RNA degradation products a chi-test was performed per locus, taking into account the number of reads in phase and the reads not in phase (*p* < 0.01). Finally, a locus was predicted as a phasiRNA if it passed the above filters and had 3 or more 21 nt windows with a number of reads in the most abundant 5 % and genome mapped reads in at least one library. All loci located in transposable elements were removed. Once the final phasiRNA-generating loci were determined, they were tested for the identification of phase-triggering microRNAs/phasiRNA-targeted transcripts using psRNATarget [60] with default parameters and a limit of expectation of 5.

### Sample preparation and expression analysis

Total RNA was isolated from 100 mg of frozen roots and nodules (21 dpi) from three biological replicates using Trizol reagent (Life Technologies) following the manufacturer’s instructions. cDNA was synthesized from 2 μg of total RNA using RevertAid™ H Minus First Strand cDNA Synthesis Kit (Fermentas). Resulting cDNAs were then diluted and used to perform qRT-PCR assays using SYBR Green PCR Master Mix (Applied Biosystems), following the manufacturer’s instructions. The sequences of oligonucleotide primers used are provided in Additional file 8: Table S5. Reactions were analyzed in a real-time thermocycler Applied Biosystem 7300. Two technical replicates were performed for each reaction. Relative expression was calculated with the “comparative Ct method” and normalized with the geometrical mean of three housekeeping genes (HSP, MDH & Ubiquitin9) [106].

## Availability of supporting data

The RNA-seq data sets supporting the results of this study are available in the Gene Expression Omnibus (GEO) database, under accession GSE67433.

## Additional files

Additional file 1: Table S1. List of the identified microRNA precursors and their corresponding mature forms. The blue, yellow and red lines represent the known miRNAs, the new isomiRs and the novel miRNAs, respectively. The “All precursors” sheet lists all the precursors identified that fulfill our criteria. The “All matures nr” sheet lists all the non-redundant mature miRNAs resulting from the “All precursors” list. The “Families” sheet lists all the miRNA families identified in our study and the corresponding number of members found. Additional file 2: Table S2. List of the miRNAs and the corresponding number of reads present in the different organs. The blue, yellow and red lines represent the known miRNAs, the new isomiRs and the novel miRNAs, respectively. The absence or presence of a “1” in the presence columns indicate the absence or the presence of the corresponding miRNA (lines) in the corresponding library (rows). Additional file 3: Table S3. List of miRNA degradome and psRNATarget-predicted targets. The blue, yellow and red lines represent the targets of the known miRNAs, the new isomiRs and the novel miRNAs, respectively. The “Degradome targets”, “Degradome targets rejected miRs”, “psRNATarget-predicted targets” and “psRNATarget target reject” sheets list the targets predicted by degradome data for the selected and rejected miRNAs, and the targets predicted by psRNATarget for the selected and rejected miRNAs, respectively. Additional file 4: Figure S1. Distribution of sequencing reads from soybean nodule libraries mapped on the soybean precursor of miRNov153. (A) Minimum free energy structure prediction of miRNov153 precursor of soybean. Heat colors represent the base-pair probabilities for each nucleotide (Blue = 0; Red = 1). The brackets show the position of miRNov153 mature and star. (B) Visualization of the mapped read distribution on the miRNov153 soybean precursor. miRNA reference sequence is at the top. The oriented grey bars represent the mapped reds. The brackets show the position of miRNov153 mature and star. Additional file 5: Table S4. List of the identified PHAS genes and their distribution in 5 plant organs. The presence of a “0” or “1” in the libraries columns indicates the absence or presence of the corresponding PHAS locus (lines) in the corresponding library (rows), respectively. The conservation column indicates the initials of the legume species in which the PHAS locus is conserved (Gma = *Glycine max*, Mtr = *Medicago truncatula*). Additional file 6: Figure S2. Venn diagram of the conservation of the PHAS genes in 3 legume species. Venn diagram of the distribution of PHAS genes based on their presence in three legumes: *Medicago truncatula* (red), *Glycine max* (yellow) and *Phaseolus vulgaris* (blue). The numbers in each Venn diagram area correspond to the numbers of *PHAS* genes encountered in the corresponding overlapping species area. Additional file 7: Figure S3. Distribution of the identified PHAS genes in 5 plant organs. Venn diagram of the distribution of PHAS gene expression in the different studied organs. Red: flower, green: leaf, yellow: root, blue: seedling and black: nodule. The numbers in each Venn diagram area correspond to the numbers of expressed phasiRNA loci encountered in the corresponding overlapping organ area. Additional file 8: Table S5. List of oligonucleotide sequences used in quantification experiments.

## Abbreviations

5’RACE: 5'-rapid amplification of cDNA ends
AGO: Argonaute
bp: Base pair
Cv: Cultivar
dsRNA: Double-strand RNA
GO: Gene Ontology
kcal: Kilocalorie
miRNA: microRNA
mol: Molar
MYB: Myeloblastosis
NB-LRR: Nucleotide Binding domain (NB) and a Leucine Rich Repeat (LRR) domain
ncRNA: Non-coding RNA
NSP: Nodulation Signaling Pathway
nt: Nucleotide
PPR: Pentatrico peptide repeat
RISC: RNA-induced silencing complex
rRNA: Ribosomal RNA
TasiRNA: Trans-acting siRNAs
tRNA: Transfer RNA
UTR: Untranslated region
