# Supplementary material for: Genome-wide identification of the Phaseolus vulgaris sRNAome using small RNA and degradome sequencing
Source: BMC Genomics. 2015 Jun 2;16(1):423. doi: 10.1186/s12864-015-1639-5 (PMC4462009; doi:10.1186/s12864-015-1639-5)
Supplement: Additional file 4: Figure S1. — Distribution of sequencing reads from soybean nodule libraries mapped on the soybean precursor of miRNov153. (A) Minimum free energy structure prediction of miRNov153 precursor of soybean. Heat colors represent the base-pair probabilities for each nucleotide (Blue = 0; Red = 1). The brackets show the position of miRNov153 mature and star. (B) Visualization of the mapped read distribution on the miRNov153 soybean precursor. miRNA reference sequence is at the top. The oriented grey bars represent the mapped reds. The brackets show the position of miRNov153 mature and star. [file 12864_2015_1639_MOESM4_ESM.pptx]

## Slide 1
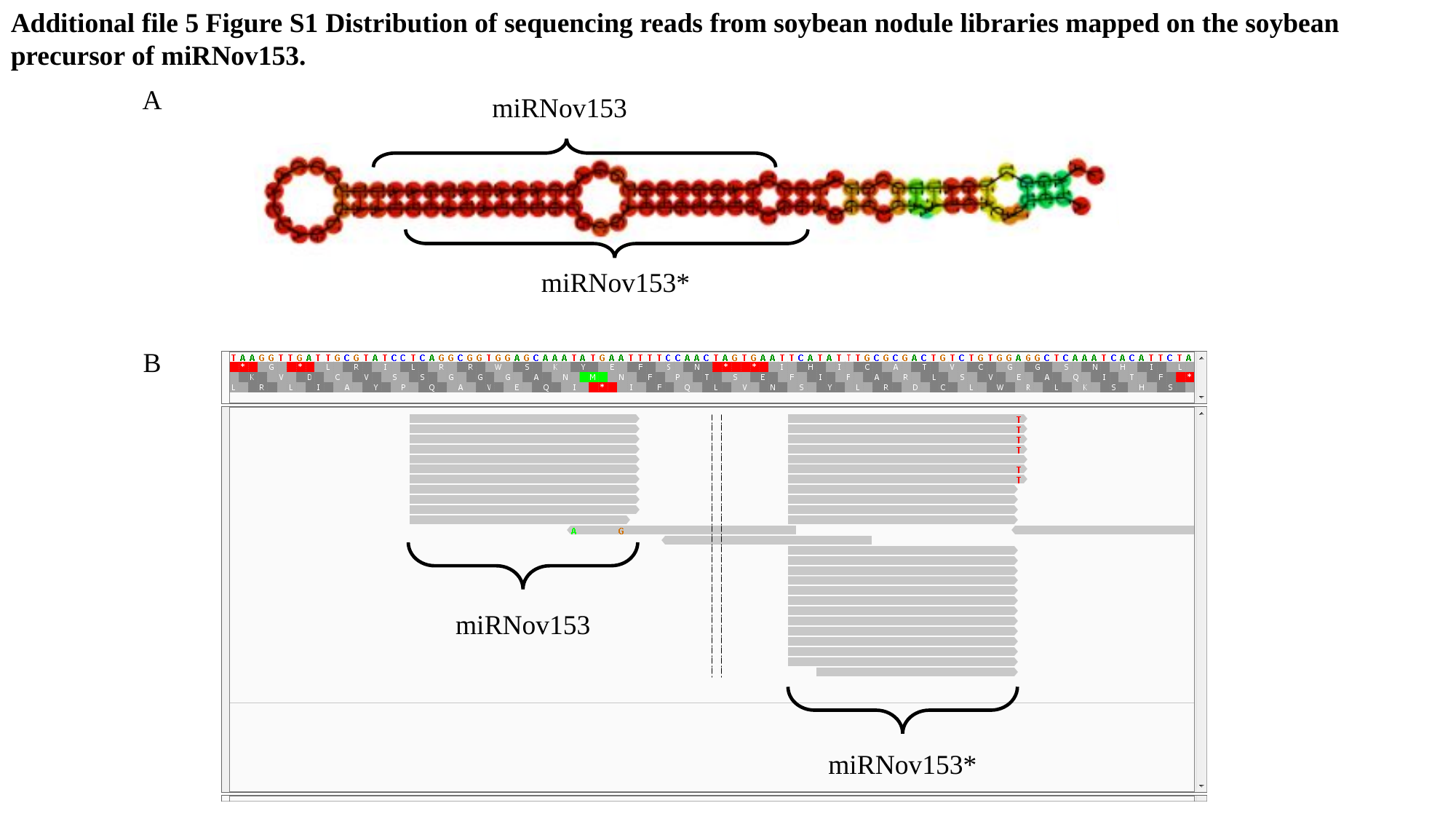

Additional file 5 Figure S1 Distribution of sequencing reads from soybean nodule libraries mapped on the soybean precursor of miRNov153.
A
miRNov153
miRNov153*
B
miRNov153
miRNov153*
