# Supplementary material for: Genome-wide identification of the Phaseolus vulgaris sRNAome using small RNA and degradome sequencing
Source: BMC Genomics. 2015 Jun 2;16(1):423. doi: 10.1186/s12864-015-1639-5 (PMC4462009; doi:10.1186/s12864-015-1639-5)
Supplement: Additional file 6: Figure S2. — Venn diagram of the conservation of the PHAS genes in 3 legume species. Venn diagram of the distribution of PHAS genes based on their presence in three legumes: Medicago truncatula (red), Glycine max (yellow) and Phaseolus vulgaris (blue). The numbers in each Venn diagram area correspond to the numbers of PHAS genes encountered in the corresponding overlapping species area. [file 12864_2015_1639_MOESM6_ESM.pptx]

## Slide 1
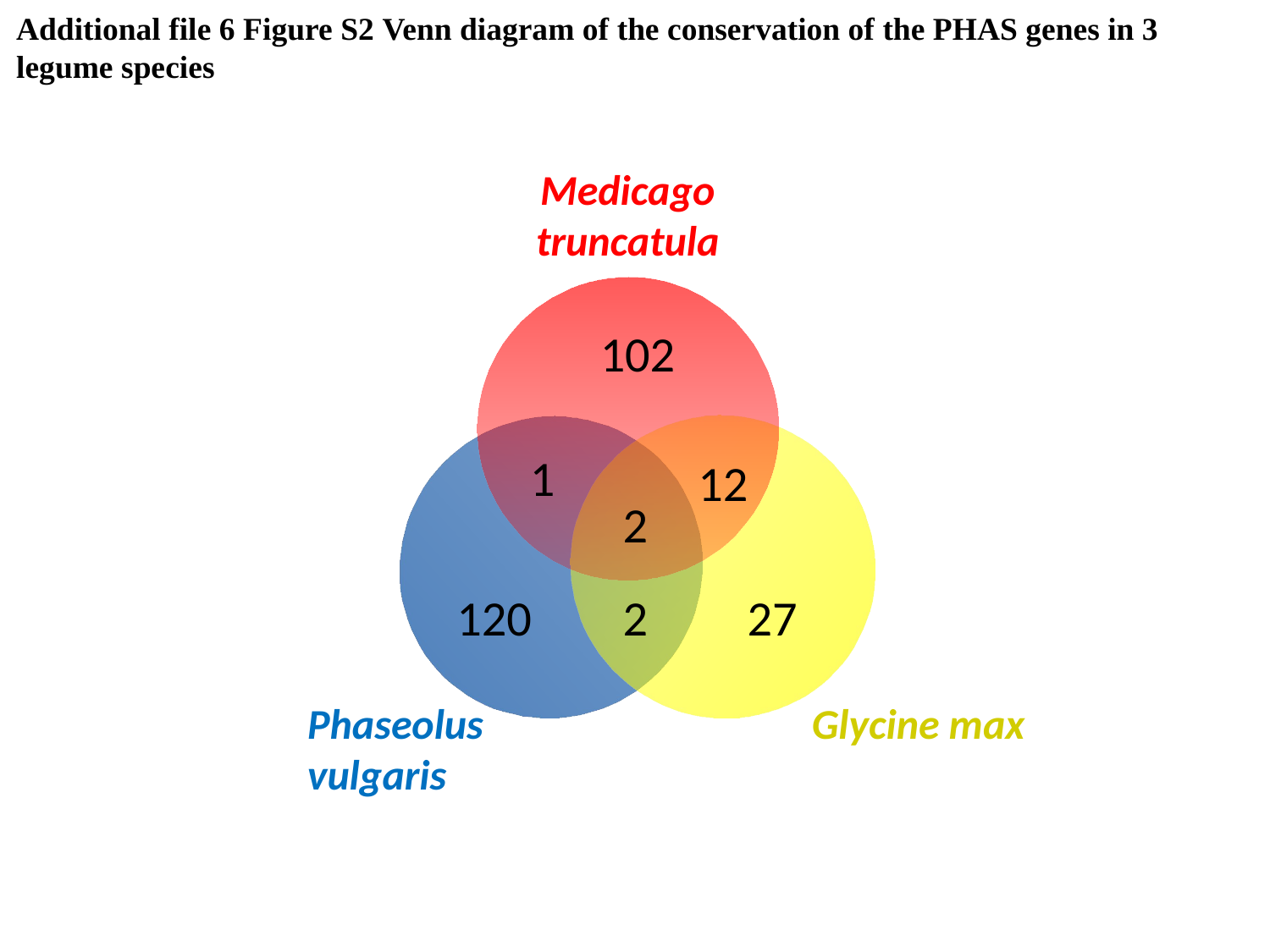

Additional file 6 Figure S2 Venn diagram of the conservation of the PHAS genes in 3 legume species
Medicago truncatula
102
1
12
2
120
2
27
Glycine max
Phaseolus vulgaris
