# Supplementary figures and images for: Genome-wide identification of the Phaseolus vulgaris sRNAome using small RNA and degradome sequencing
Source: BMC Genomics. 2015 Jun 2;16(1):423. doi: 10.1186/s12864-015-1639-5 (PMC4462009; doi:10.1186/s12864-015-1639-5)

## Slide 1
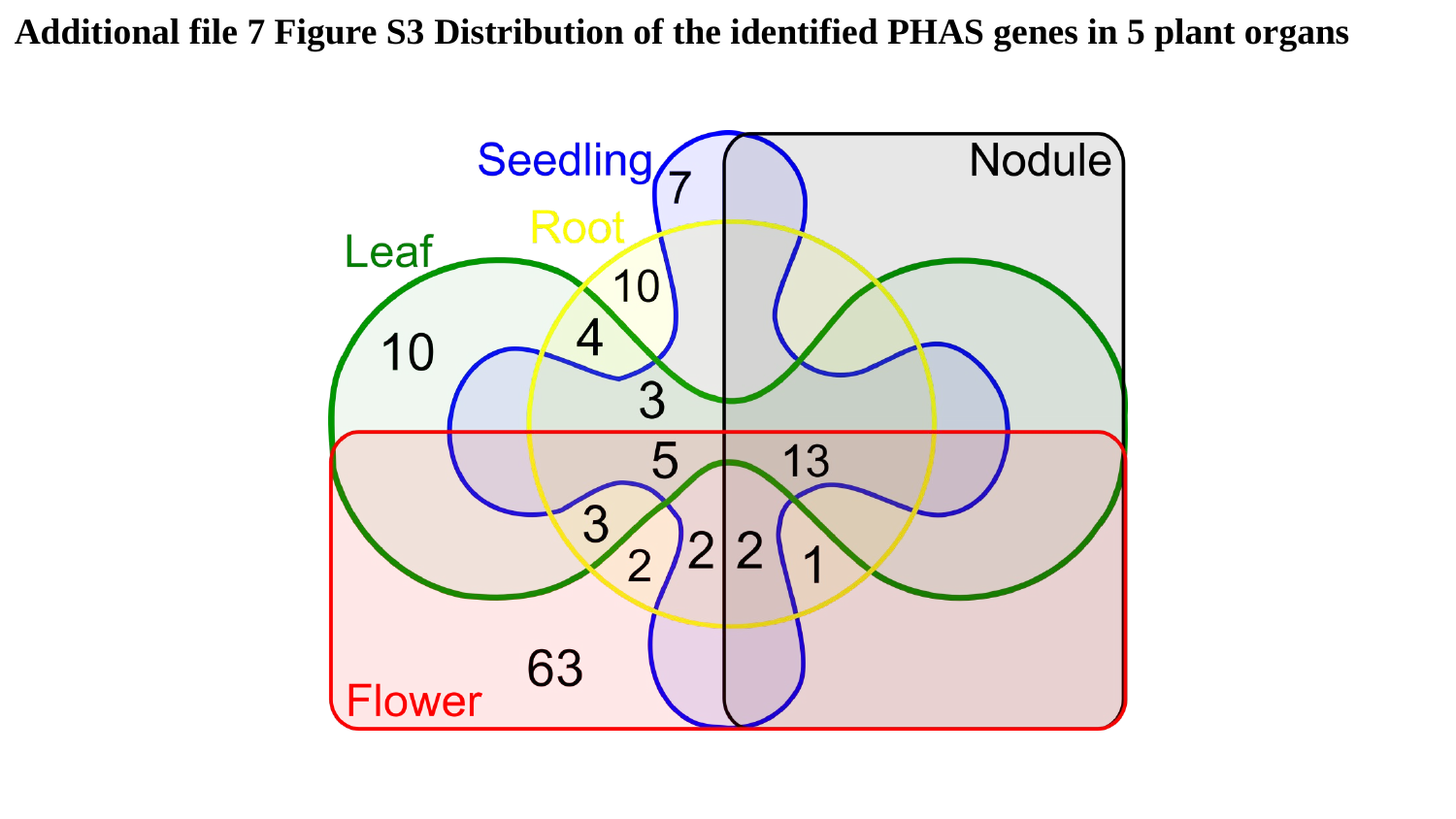

Additional file 7 Figure S3 Distribution of the identified PHAS genes in 5 plant organs

Supplement: Additional file 7: Figure S3. — Distribution of the identified PHAS genes in 5 plant organs. Venn diagram of the distribution of PHAS gene expression in the different studied organs. Red: flower, green: leaf, yellow: root, blue: seedling and black: nodule. The numbers in each Venn diagram area correspond to the numbers of expressed phasiRNA loci encountered in the corresponding overlapping organ area. [file 12864_2015_1639_MOESM7_ESM.pptx]
